# The neuroprotective and neural circuit mechanisms of acupoint stimulation for cognitive impairment

**DOI:** 10.1186/s13020-023-00707-x

**Published:** 2023-01-20

**Authors:** Zichen Zhang, Liuyi Chen, Yi Guo, Dan Li, Jingyu Zhang, Ling Liu, Wen Fan, Tao Guo, Siru Qin, Yadan Zhao, Zhifang Xu, Zelin Chen

**Affiliations:** 1 Research Center of Experimental Acupuncture Science, Tianjin University of Traditional Chinese Medicine, Tianjin, 301617 People’s Republic of China; 2 First Clinical College, Hubei University of Chinese Medicine, Wuhan, 430065 People’s Republic of China; 3 School of Acupuncture and Moxibustion and Tuina, Tianjin University of Traditional Chinese Medicine, Tianjin, 301617 People’s Republic of China; 4 National Clinical Research Center for Chinese Medicine Acupuncture and Moxibustion, Tianjin, 300381 People’s Republic of China; 5 Department of Rehabilitation Physical Therapy Course, Faculty of Health Science, Suzuka University of Medical Science, Suzuka City, 5100293 Japan

**Keywords:** Acupoint stimulation, Acupuncture, Cognitive impairment, Dementia, Neuroprotection, Neural circuit

## Abstract

Cognitive impairment is a prevalent neurological disorder that burdens families and the healthcare system. Current conventional therapies for cognitive impairment, such as cholinesterase inhibitors and N-methyl-d-aspartate receptor antagonists, are unable to completely stop or reverse the progression of the disease. Also, these medicines may cause serious problems with the digestive system, cardiovascular system, and sleep. Clinically, stimulation of acupoints has the potential to ameliorate the common symptoms of a variety of cognitive disorders, such as memory deficit, language dysfunction, executive dysfunction, reduced ability to live independently, etc. There are common acupoint stimulation mechanisms for treating various types of cognitive impairment, but few systematic analyses of the underlying mechanisms in this domain have been performed. This study comprehensively reviewed the basic research from the last 20 years and found that acupoint stimulation can effectively improve the spatial learning and memory of animals. The common mechanism may be that acupoint stimulation protects hippocampal neurons by preventing apoptosis and scavenging toxic proteins. Additionally, acupoint stimulation has antioxidant and anti-inflammatory effects, promoting neural regeneration, regulating synaptic plasticity, and normalizing neural circuits by restoring brain functional activity and connectivity. Acupoint stimulation also inhibits the production of amyloid β-peptide and the phosphorylation of Tau protein, suggesting that it may protect neurons by promoting correct protein folding and regulating the degradation of toxic proteins via the autophagy-lysosomal pathway. However, the benefits of acupoint stimulation still need to be further explored in more high-quality studies in the future.

## Introduction

Cognitive impairment is a common neurodegenerative disease. In the past two decades, dementia’s implication as a syndrome of global cognitive impairment has gained widespread recognition. More than 50 million individuals worldwide are affected by dementia, which has become the leading cause of disability and dependence among the elderly [1]. Alzheimer’s disease (AD) and vascular dementia (VD) are the two main causes of dementia [2, 3]. Mild cognitive impairment (MCI) is gaining attention as an intermediate stage between normal cognition and dementia, with a prevalence of 10–20% among those over the age of 65 [4]. Loss of neurons and synapses are the main pathological features of cognitive impairment, which leads to impairment of basic functions such as cognition and gradual loss of autonomous living ability [5]. This places a heavy burden on the patients’ families and the healthcare system. It is estimated that the global economic cost of dementia could rise to US$2 trillion by 2030 [6]. Drug therapy, including cholinesterase inhibitors (donepezil, galantamine, and rivastigmine) and N-methyl-d-aspartate receptor (NMDAR) antagonists (i.e., amantadine), is the currently available standard treatment for dementia. However, these drugs cannot prevent or reverse the progression of dementia and are associated with gastrointestinal, cardiovascular, and sleep disorders, as well as other side effects [7, 8]. Exploring new, safe, and effective therapies is a crucial aspect of neuroscience.

Traditional Chinese medicine (TCM), which includes acupuncture, electroacupuncture (EA), moxibustion, and other therapies, places a high priority on acupoint stimulation. These therapies have been demonstrated to be effective in treating multiple neurological disorders [9–11]. Acupuncture has been recommended by WHO as an effective complementary and alternative therapy in the treatment of VD [12], a recently published overview of systematic reviews of acupuncture for 77 diseases found that acupuncture significantly reduced the severity of VD symptoms [13], and it also shows potential in the treatment of other types of cognitive impairment. A systematic review and meta-analysis of 13 randomized controlled trials (RCTs) with 750 cases showed that acupuncture could help AD patients with cognitive impairment and that it was more effective than traditional Western medicine on the Mini-Mental State Examination (MMSE), the Activity of Daily Living (ADL), and the AD Assessment Scale-Cognitive scales, with no serious side effects [14]. Another study analyzed and compared 15 RCTs concerning acupuncture treatment for MCI, involving 1051 subjects, and found that compared with the control group, acupuncture treatment resulted in better clinical efficacy rates, as well as improved MMSE, Montreal Cognitive Assessment, clock-drawing task, and ADL scores, indicating that acupuncture is beneficial for improving cognitive function in elderly people with MCI [15]. Acupoint stimulation is also effective in improving cognitive deficits in memory, language, and executive function caused by surgery [16], chemotherapy [17], depression [18], and schizophrenia [19]. In addition, acupoint stimulation alone has better efficacy than Western medicine in improving patients' cognitive function and living ability, and synergistic therapy with Western medicine or Chinese herbal medicine may be superior to Western medicine alone [20–25].

Clinically, acupoint stimulation has the potential to treat symptoms of common cognitive disorders such as memory impairment, language impairment, executive dysfunction, and decreased functional capacity, among others. Taken together, this therapeutic strategy pays attention to symptoms when selecting acupoints. Similar acupoints have been selected, such as *Baihui* (GV20), *Sishencong* (EX-HN1), *Fengchi* (GB20), *Shuigou* (GV26), *Shenting* (GV24), *Neiguan* (PC6), *Zusanli* (ST36), *Sanyinjiao* (SP6), *Shenmen* (HT7), *Taixi* (KI3), *Shenshu* (BL23), *Fenglong* (ST40), *Taichong* (LR3), *Dazhui* (GV14), *Xuanzhong* (GB39), etc. It is suggested that there are common mechanisms of acupoint stimulation in the treatment of various forms of cognitive impairment. According to preliminary research, in models of cognitive impairment, acupoint stimulation can improve spatial learning and memory. In recent years, however, few systematic analyses of the underlying mechanisms in this domain have been conducted. Therefore, we reviewed the basic research from the past two decades to evaluate the common mechanisms of acupoint stimulation for the treatment of various forms of cognitive impairment and to provide new evidence for its clinical application.

## Methods

### Search strategy

For this study, the PubMed, Web of Science, and Embase databases were searched for the articles that were published between August 2001 and December 2022. The keywords included “acupuncture”, “electroacupuncture”, “moxibustion”, “transcutaneous acupoint electrical stimulation”, “neurocognitive disorders”, “cognitive defect”, “cognitive impairment”, and their related terms. A total of 1702 articles in English were identified.

### Study selection

**Fig. 1:**
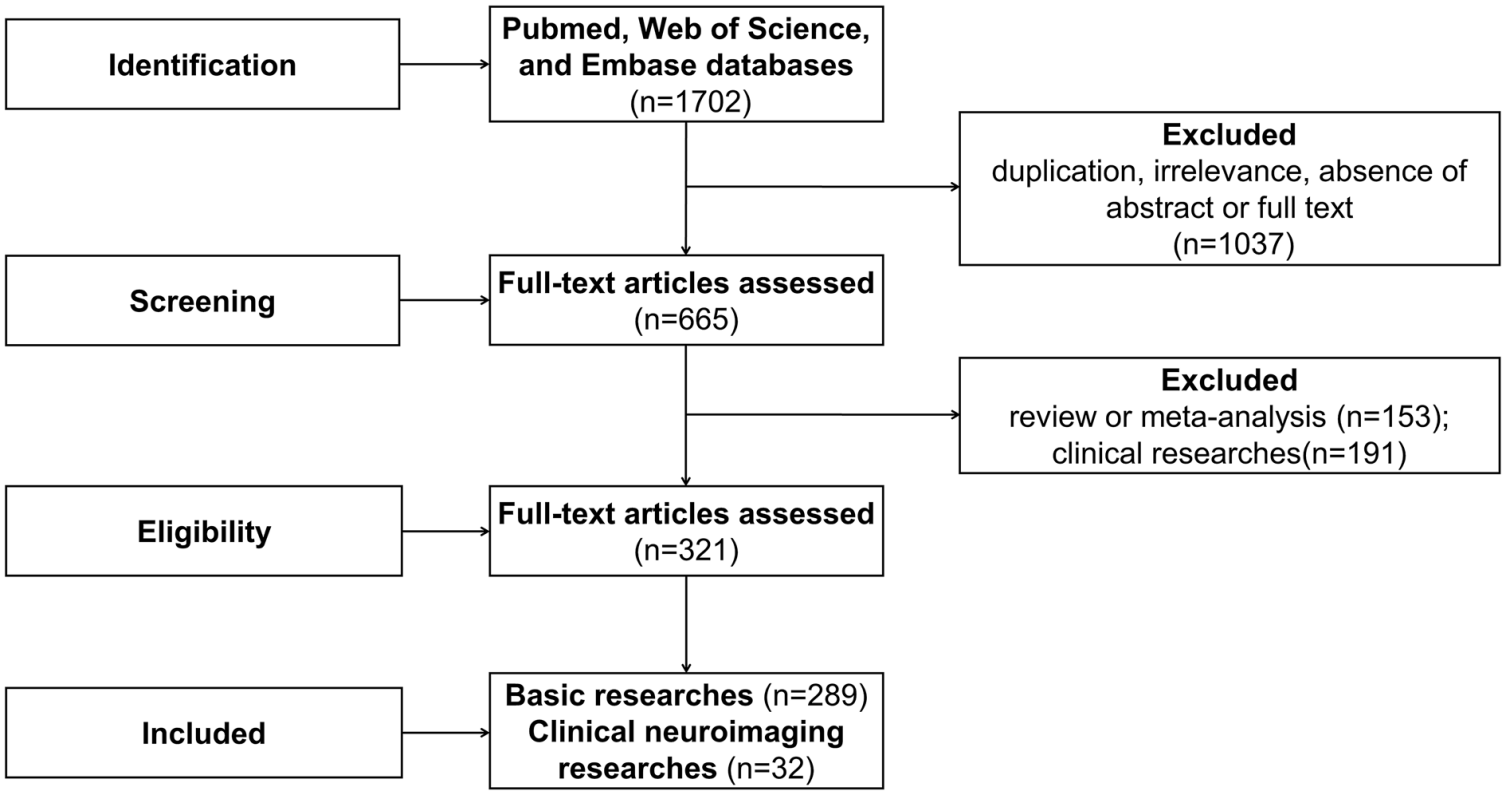
Flow chart of the search strategy and process

The following inclusion criteria were used for screening of the selected articles: stimulation methods included manual acupuncture (MA), EA, moxibustion, and transcutaneous acupoint electrical stimulation, and the main diseases studied included AD, VD, MCI, and cognitive impairment caused by various pathological factors. The 1702 articles identified by the search engine were then manually screened for those that met our inclusion criteria based on title and abstract. This led to the exclusion of 1037 articles due to duplication, irrelevance, lack of abstract, or unavailability of full text while the remaining 665 articles, including 289 basic studies, 223 clinical studies, and 153 reviews or meta-analyses were included. There were a total of 289 basic studies that constituted the underlying research. The 32 neuroimaging studies were selected from the 223 clinical studies because of their potential relevance to neural circuits. There were a total of 191 excluded clinical studies and 153 reviews or meta-analyses. Finally, 321 studies were analyzed. The flow chart of the search process is shown in Fig. 1.

### Data extraction

Two authors have independently studied the titles, abstracts, and full texts of the retrieved articles. As a result, 321 articles were finally included, followed by data collection based on the predetermined criteria. Information from 56 recent representative studies is summarized in Table 1. Any disagreements were resolved through discussion among the authors.

**Table 1:** Neuroprotective and neural circuit mechanisms of acupoint stimulation treating cognitive impairment

References	Model/Subjects	Intervention methods	Acupoints	Acupoint stimulation parameters	Effect measurements	Biochemical measurements
[31]	d-galactose and Aβ_1-40_ injection rats	MA	GV24, GB13	Twisting at 80 ± 5 times/min, 180° ± 5°, 2 min, with 1 min rest, 15 min, 6 days a week, 4 weeks	The escape/avoidance training: the number and duration of electric shock↓	Serum: ChAT↑, AChE↓, SOD↑, MDA↓, ROS↓; Hippocampus: neurons number↑, cells intercellular space↓, TUNEL indexes↓, Bcl-2↑, Bax↓, CYC↓, caspase3↓, caspase 9↓, Bcl-2/Bax↑
[32]	Aβ_1-40_ injection rats	EA	GV20, BL23	20 Hz, 30 min, 6 days a week, 4 weeks	MWM test: escape latency↓, platform cross number↑, Time spent in the target quadrant↓	CA1: cell apoptosis↓; Hippocampus: Bcl-2 protein↑, Bax protein↓, synapsin-1↑, Notch1 mRNA↓, Hes1 mRNA↓
[36]	MCAO/R rats	EA	GV20, GV24	1/20 Hz, 30 min, 10 days	Cerebral infarct volumes↓, MWM test: the latency and route length↓, platform cross number↑	TUNEL indexes↓, NF-κB protein↓, p-IκB↓, Fas↓, Bax↓
[37]	Aβ_1–42_ injection rats	Moxibustion	GV20, BL23	Pre-moxibustion group: 15 min, 6 days a week, 8 weeks + 2 weeks; moxibustion group: 15 min, 6 days a week, 2 weeks	MWM test: escape latency↓, platform cross number↑, time spent in the target quadrant↑	CA1: cell apoptosis (Pre-moxibustion group was superior to moxibustion group)↓, cell membrane ruptured↓
[40]	BCCAO rats	EA	GV20, GV14, BL23	4 Hz, 2 mA, 20 min, 30 days	–	CA1: p53↓, Noxa↓
[42]	APP/PS1 mice	EA	GV20, GV29, GV26	1 Hz, 1 mA, 20 min	MWM test: escape latency↓, platform cross number↑	Hippocampus: APP↓, p-JNK↓, p-MKK7↓, c-Jun↓
	APP/PS1 mice	EA + SP600125	GV20, GV29, GV26	1 Hz, 1 mA, 20 min	MWM test: escape latency↑, platform cross number↓	Hippocampus: APP↓, P-JNK↓, p-MKK7↓, c-Jun↓
[44]	2VO rats	MA	GV20, ST36	6 days per week, 2 weeks	MWM test: escape latency↑, swimming distance↑, time spent in the target quadrant↓	Hippocampus: ROS↓, TUNEL indexes↓, Trx-1↑, TrxR-1↑, p-ASK1↓, p-JNK↓, p-p38↓, cleaved-caspase3↓
	2VO rats	Trx-1 siRNA + MA	GV20, ST36	6 days per week, 2 weeks	MWM test: escape latency↑, swimming distance↑, time spent in the target quadrant↓	Hippocampus: ROS↑, TUNEL indexes↑
[45]	APP/PS1 mice	EA + MA	EA: GV20, GV29; MA: GV26	EA: 1 Hz, 1 mA, 20 min, MA: fast pricking, 4 weeks	MWM test: escape latency↓, Average swimming speed↑, Platform crossing frequency↑, time spent in the target quadrant↑	Hippocampus: APP↓, BACE1↓, p-PKA/total-PKA↑
[49]	D-galactose and Aβ_1-40_ injection rats	EA	GV24, GB13	30 Hz, 1 mA, 30 min, 6 days a week, 4 weeks	OFT: duration spent in the central zone↓, frequency of crossing↑; MWM test: escape latency↓, escape distance↓, latency to cross the platform for the first time↓, frequency of platform crossings↑, time spent in the target quadrant↑	Hippocampus: Aβ↓, p-tau (s396)↓, p-tau (s404)↓
[52]	APP/PS1 mice	EA + MA	EA: GV20, GV29; MA: GV26	EA: 2 Hz, 1 mA, 20 min, MA: fast pricking, once every other day for 28 days	MWM test: escape latency↓, time spent in the target↑, platform cross frequency↑	Hippocampus: glucose metabolism↑, p-Tau (Ser199, Ser202)↓, p-AKT (Ser473)↑, p-GSK3β (Ser9)↑
[53]	SAMP8 mice	MA	CV17, CV12, CV6, SP10, ST36	30 s	Tightrope test: success rate↑; MWM test: the number of platform-site crossovers↑, the latency to first target-site crossover↓, percentage of time spent in the middle annulus↑	Hippocampus:CA3↑, DG↑, SOD↑, GSH-Px↑, superoxide anion↓, protein carbonyl concentrations↓, HSP84↑, HSP86↑
[54]	APP/PS1 mice	EA	GV20	1/20 Hz, 30 min, 5 days a week, 4 weeks	MWM test: escape latency↓, escape distance↓, frequency of platform crossings↑ time spent in the target quadrant↑, discrimination ratio↑	Cortex and Hippocampus: glucose metabolism↑, GLUT1↑, GLUT3↑, Aβ↓, AMPK↑, AKT↑, p-mTOR/total-mTOR↓
[55]	MCAO rats	EA	GV20, GV24	1–20 Hz, 2 mA, 30 min, 8 days	Neurological scores↓, cerebral infarction volume↓, MWM test: escape latency↓, platform crossings frequency↑	Cell membrane ruptured↓, mitochondria damaged↓, PI3K↑, mTOR↑, Beclin-1↑, p53↓, p-Akt↑
[56]	5xFAD mice	EA	GV24, GB13	2 Hz, 0.3 mA, 15 min, 5 days a week, 4 weeks	MWM test: escape latency↓, platform crossings frequency↑, time spent in the target↑; Contextual and Cued test: freezing index↑	Fl-APP↓, CTFs↓, Aβ positive area↓, plaque size↓, microglia activation↓, insoluble LC3B-II↓, insoluble SQSTM1↓, LC3B^+^ CTSD↑, LAMP1↑, total TFEB↑, nuclear TFEB↑, p-TFEB (S142)↑, LC3B ^+^/Aβ1-42 ^+^↑, APP-associated SQSTM1↓, 6E10 ^+^ plaques↓, plaque-associated LAMP1↓, p-MTOR↓, RPS6↓, p-AKT in the PFC↓, p-MAPK1 in the hippocampus↓
	5xFAD mice	EA + CQ	GV24, GB13	2 Hz, 0.3 mA, 15 min, 5 days a week, 4 weeks	MWM test: platform crossings frequency↓, time spent in the target↓, Contextual and Cued test: freezing index↓	Insoluble LC3B-II↑, insoluble SQSTM1↑, LAMP1↓, Fl-APP↑
	5xFAD mice	EA + AAV-sh-*Tfeb*	GV24, GB13	2 Hz, 0.3 mA, 15 min, 5 days a week, 4 weeks	MWM test: platform crossings frequency↓	CTFs↑
[58]	MCAO rats	MA	GV20, GV26, GV14, GV16	20 min, 15 days	MWM test: escape latency↓, platform cross number↑	TUNEL indexes↓, ATP↑, SOD↓, NO↓, iNOS↓, ROS↓, TOMM40↓, TIMM17A↓, Aβ↓, APP↑, COX↑
[59]	LI/R rats	EA pretreatment	GV20, GB34, LR3, ST36, SP10	2/15 Hz, 1 mA, 30 min	Survival rate↑, MWM test: escape latency↓, distance to platform↓, time spent in the target quadrant↑, platform cross number↑	CA1: number of normal cells↑, TUNEL ^+^ cells↓, microglia activation↓
[60]	Aβ1-42 injection rats	EA	GV20, KI1	2/15 Hz, 1 mA, 30 min, 4 weeks	MWM test: escape latency↓, time spent in the target quadrant↑	Hippocampus: ROS↓, 8-OH-dG↓, MDA↓, T-AOC↑, NOX2↓, swelling of neurons↓
[61]	AF64A injection rats	LA	HT7	Wavelength of 405 nm, 10 min, 2 weeks	MWM test: escape latency↓, retention time↑	Hippocampus: ROS↓, MDA↓, CAT activity↑, SOD activity↑, AChE activity↓
[69]	2VO rats	MA	GV20, ST36	Twisting < 90°, > 120 times per min, 30 s	MWM test: the escape latency↓, swimming distance↓, time spent in the target quadrant↑; infarct volume↓, neuronal cell loss↓	O_2_^−^↓, NADPH oxidase activity↓, gp91phox protein↓, p47phox protein↓
	2VO rats	MA + TBCA	GV20, ST36	Twisting < 90°, > 120 times per min, 30 s	Infarct volume↑	O_2_^−^↑
	Gp91phox KO mice	MA	GV20, ST36	Twisting < 90°, > 120 times per min, 30 s	MWM test: the escape latency↑, swimming distance↑, time spent in the target quadrant↓	–
[71]	2VO rats	MA	GV20, ST36	2 weeks, suspend on day 7	CBF↑, MWM test: the escape latency↓, time spent in the target quadrant↓	CA1: injured neuron cell↓, ROS↓; Hippocampus: Nrf2 nuclear translocation↑, Nrf2↑, HO-1 protein↑, NQO1 protein↑, microglia activation↓, neuron death↓
	2VO mice (Nrf2 KO)	MA	GV20, ST36	2 weeks, suspend on day 7	MWM test: the escape latency↑, time spent in the target quadrant↑	CA1: microglia activation↑, neuron death↑
[76]	APP/PS1 mice (mild AD)	EA	GV20, GV24	1/20 Hz, 1 mA, 30 min, 3 days a week, 16 weeks	MWM test: escape latency↓, platform cross number↑	Parietal association cortex: number and area fraction of Aβ↓; colocalization of iNOS-Iba1↓, IL-1β-Iba1↓, CD206-Iba1↑, Arg1-Iba1↑; iNOS mRNA↓, IL-1β mRNA↓, CD206 mRNA↑, Arg1 mRNA↑; Entorhinal cortex: colocalization of Arg1-Iba1↑; iNOS mRNA↓, IL-1β mRNA↓, CD206 mRNA↑, Arg1 mRNA↑
	APP/PS1 mice (moderate AD)	EA	GV20, GV24	1/20 Hz, 1 mA, 30 min, 3 days a week, 16 weeks	MWM test: escape latency↓, platform cross number↑	Parietal association cortex: number of Aβ↓; colocalization of iNOS-Iba1↓; Entorhinal cortex: number of Aβ↓; colocalization of iNOS-Iba1↓, Arg1-Iba1↑; IL- 1β mRNA↓, CD206 mRNA↑
[80]	BCCAO Mongolian gerbils	BVA	ST36	0.1 μg/g, 4 times every other day	Y-Maze task: spontaneous alternation↑	CA1: neuronal cell death↓, microglia activation↓; Hippocampus: TLR4↓, CD14↓, TNF-α↓, iNOS↓, Bax↓, BDNF↑, p-ERK↑
[81]	2VO rats	MA	GV20, ST36	6 days per week, 2 weeks	MWM test: escape latency↓, time spent in the target↑	Hippocampus and plasma: IL-6↓, TNF-α↓; Hippocampus: TLR4↓, TLR4 ^+^ microglia↓, MyD88↓, p-NF-κB↓, p65↓, miR-93↓
[85]	SAMP8 mice	EA	GV20, ST36	2 or 10 Hz, 1 mA, 10 min, 2 weeks	MWM test: escape latency↓, travel distance↓, platform cross frequency↑	CA1 and CA3: morphological and structural abnormalities of neurons↓, TUNEL indexes↓; Serum: IL-1β↓, IL-6↓, IL-18↓, TNF-α↓; Hippocampus: NLRP3↓, ASC↓, caspase-1↓, GSDM-D↓, IL-1β↓, IL-18↓ (10 Hz was superior to 2 Hz)
[86]	2VO rats	MA	GV20, ST36	Twisted 2 times per sec, 30 s, 2 weeks, suspend on day 7	MWM test: escape latency↓, total distance↓, the time spent in the target quadrant↓	CA1: cell apoptosis↓, ROS↓, 8-OHdG↓, SOD↑, TXNIP↓, NLRP3↓, caspase-1↓, IL-1β↓
[90]	2VO rats	MA	GV20, ST36	10 min, 6 days per week, 2 weeks	MWM test: escape latency↓, the time spent in the target quadrant↓	Hippocampus: injured neurons↓, TNF-α↓, IL-6↓, microglia activation↓, α7nAChR ^+^ neurons↑, p-JAK2↑, p-STAT3↑
	2VO rats	MA + α-BGT	GV20, ST36	10 min, 6 days per week, 2 weeks	MWM test: escape latency↑, the time spent in the target quadrant↓	Hippocampus: injured neurons↑, TNF-α↑, IL-6↑
[93]	MCAO/R rats	EA	GV20, GV24	2/20 Hz, 0.2 mA, 30 min, 7 days	MWM test: escape latency↓, platform cross number↑, infarct volume↓	Peri-infarct CA1 and sensorimotor cortex: ED1^+^ cells↓, GFAP^+^ cells↓, IL-1β↑, IL-10↓, P2X7R^+^/ED1^+^ cells↓, P2X7R ^+^/GFAP^+^ cells↓, P2Y1R^+^/ED1^+^ cells↓, P2Y1R^+^/GFAP^+^ cells↓
[94]	MID rats	MA	ST36	Twisted 2 times/s for 30 s, 6 days a week, 2 weeks	MWM test: escape latency↓	Pyramidal neurons number in the CA1↑
[95]	SAMP8 rats	MA	CV17, CV12, CV6, SP10, ST36	rotated twice a second, 30 s	MWM test: escape latency↓, retention time↓	Neurons number in the CA3 and DG↑
[96]	SAMP8 rats	MA + NSCs transplantation	CV17, CV12, CV6, SP10, ST36	CV17, CV12, CV6, ST36: twisting reinforcing methods; SP10: twisting reducing method; 15 days, suspended on day 7	MWM test: escape latency↓, retention time↓, platform cross frequency↑	Hippocampus: bFGF↑, EGF↑, BDNF; DG and in vitro: NeuN ^+^/BrdU^+^ NSCs↑, GFAP ^+^/BrdU^+^ NSCs↑
[97]	Brain irradiation rats	EA	GV20, ST36	2/15 Hz, 2–3 mA, from 3 days before irradiation to 2 weeks post-irradiation	NORT: novel place exploration ratio↑, stool droppings↓, latency↓	Hippocampus: activated microglia↓, BDNF↑; DCX ^+^ cells in the SGZ↑
[98]	SAMP8	MA	CV17, CV12, CV6, SP10, ST36	CV17, CV12, CV6, ST36: twisting reinforcing methods; SP10: twisting reducing method, 15 days, suspended on day 8	MWM test: escape latency↓, retention time↓	BrdU^+^ cells in the DG and subfield of LV-CC↑
[102]	5xFAD mice	EA	GV20, GV24	2/20 Hz, 1 mA, 30 min, 5 days a week, 4 weeks	NLRT: location exploration↑	MS/VDB and DG: NAA/Cr↑, Cho/Cr↑, the number of M1 mAChR^+^ cells↑; the neurons were arranged more neatly, with darker and more obvious Nissl bodies; MS/VDB: AChE↓, VAChT↑; DG: ChAT↑, Aβ fraction ratio↓; Hippocampus: DCX^+^ cells↑, Neuro-D1^+^ cells↑
	5xFAD mice	EA + hM4Di	GV20, GV24	2/20 Hz, 1 mA, 30 min, 5 days a week, 4 weeks	–	MS/VDB and DG: Cho/Cr↓, the number of M1 mAChR^+^ cells↓; Hippocampus: DCX^+^ cells↓, Neuro-D1^+^ cells↓
[103]	BCAS mice	EA	GV20, GV14	2 Hz, 20 min, 7 days	MWM test: latency to locate platform↓, step through latency↓	CC: fluoromyelin staining↓, MBP↓, BrdU ^+^ cells↑, BrdU ^+^/NG2 ^+^ cells↓, BrdU ^+^/CNPase ^+^ cells↑, NG2↓, PDGFRα↓, CNPase↑, Figf↑, Mdk↑, NT4/5↑, NT4/5 ^+^/GFAP cells↑, p-TrkB ^+^ cells↑, p-TrkB ^+^/PDGFRα ^+^ cells↑, p-TrkB ^+^/ CNPase ^+^ cells↑, p-CREB ^+^ cells
	BCAS mice	EA + ANA-12	GV20, GV14	2 Hz, 20 min, 7 days	MWM test: latency to locate platform↑	BrdU ^+^/NG2 ^+^ cells↑, BrdU ^+^/CNPase ^+^ cells↓
[111]	MCAO/R rats	EA	GV20, GV24	2/10 Hz, 1–3 mA, 30 min, 7 days	Cerebral infarction↓; MWM test: escape latency↓, retention time↓, platform cross number↑	Hippocampus: vacuolization of mitochondria in the axons↓, BDNF↑, TrkB↑, PSD-95
[114]	Aβ_1-42_ injection rats	EA	GV20, BL23	2 or 30 or 50 Hz, 1 mA, 15 days, suspended on day 7	MWM test: escape latency↓, time spent in the target↑, platform cross number↑	Synaptic curvatures↑, synaptic cleft width↓, postsynaptic density↑, GSK-3β↓, p-GSK-3β (Tyr216)↓, APP↓, p-GSK-3β (Ser9)↑, Aβ_1–40_↓ (50 Hz > 30 Hz > 2 Hz)
[116]	MCAO rats	EA	GV20, GV24	1/20 Hz, 30 min, 7 days	Infarct volume↓, MWM test: swimming duration and distances↓	Dendritic spines density↑, Cdc42↑, Rac1↑, F-actin↑, RhoA↓
[121]	MCAO rats	EA	EX-HN3, GV20	2 Hz, 1 mA, 10 min, 14 days	MWM test: latency↓, time spent in the target↓; NORT: recognition index↑	Hippocampus and PFC: BDNF↑, TrkB↑, NMDAR1↑, AMPAR↑, GABA_A_R↑, CaMKII↑, NeuN↑, PSD-95↑
[123]	Cerebral multi-infarction rats	MA	ST36	Twisting two spins per second, 30 s, 6 days a week, 2 weeks	Radial arm maze test: working memory errors↓, reference memory errors↓	fEPSP slope in the DG↑; p-ERK in the CA1 and DG; Hippocampus: PDE activity↓, cAMP↑, PKA activity↑, p-CREB↑
	Cerebral multi-infarction rats	MA + H89	ST36	Twisting two spins per second, 30 s, 6 days a week, 2 weeks	MWM test: escape latency↑, time spent in the target↓	p-CREB↓
[127]	SAMP8	EA	GV20, ST36	10 Hz, 1 mA, 30 min, 7 days	Y Maze Spontaneous Alternation Test: correct spontaneous alternation rate↑; MWM test: escape latency↓, time spent in the target↑, platform cross number↑	CSF: Orexin A↓, Glutamate↓; Hippocampus and lateral hippocampus: Orexin A↓; Hippocampal CA1 and CA3: the pyramidal cells were orderly arranged, the metabolism of Nissl bodies↑; Hippocampus: cAMP↑, pPKA↑, PKA↑, pCREB↑, CREB↑, GluN1↑, GluN2A↑, GluA2↑, SYP↑, and PSD-95↑, Orenxin A↓, synapses↑; the mitochondrial bilayer membrane was clear, the internal cristae was orderly arranged, the myelin sheaths were intact, dense, and highly layered, synaptic structures were relatively complete and normal
[128]	2VO rats	MA	MA1: GV20, ST36; MA2: GV20, GV24; MA3: ST36, SP10	Twisting < 90°, 120 times/min, 30 s, 6 days a week, 2 weeks	MWM test: escape latency↓, swimming distance↓, time spent in the target↑ (MA1 was superior to MA2 and MA3)	–
	2VO rats	MA	GV20, ST36	Twisting < 90°, 120 times/min, 30 s, 6 days a week, 2 weeks	–	fEPSP slope in the PP-DG↑, dopamine↑, DOPAC↑, HVA↑, epinephrine↑, D1R↑, D5R↑
	2VO rats	MA + SCH23390	GV20, ST36	Twisting < 90°, 120 times/min, 30 s, 6 days a week, 2 weeks	MWM test: escape latency↓, swimming distance↑, time spent in the target↓	fEPSP slope↓
	2VO rats	MA + SKF38393	GV20, ST36	Twisting < 90°, 120 times/min, 30 s, 6 days a week, 2 weeks	–	fEPSP slope↑
[129]	2VO rats	MA	GV20, ST36	10 min, 6 days a week, 2 weeks	–	fEPSP slope in the PP-DG↑, NE↑, β1-AR↑, β1-AR ^+^/NeuN ^+^ cells↑
	2VO rats	MA + propranolol	GV20, ST36	10 min, 6 days a week, 2 weeks	–	fEPSP slope in the PP-DG↓
	2VO rats	MA + atenolol	GV20, ST36	10 min, 6 days a week, 2 weeks	–	fEPSP slope in the PP-DG↓
[130]	2VO rats	MA	GV20, ST36	10 min, 12 days, suspended on day 7	NORT: recognition index↑; RAM test: working memory errors↓, new entries number↑	LTP of population spike in the PP-DG↑, DBH↑, DBH activity↑
[133]	MCAO rats	EA	GV20, GV24	20 Hz, 30 min, 7 days	Infarct volume↓, MWM test: latency↓, route length↓, platform cross number↑	CA1: Glu↓, NMDAR2A↑, NMDAR2B↓, intracellular Ca^2 +^↓
[134]	SCO rats	MA	MA1:GV20; MA2: TE4	5 min	PAT: latencies to enter the dark compartment↑ (MA1); MWM test: latency↓, percentages of time↑ (MA1)	CA1: ChAT↑, BDNF↑, CREB↑, CHT1↑, VAChT↑ (MA1)
[136]	POCD rats	EA	GV20, PC6, LI4	2/100 Hz, 4 mA, 30 min, 7 days	MWM test: escape latency↓, platform cross number↑	Hippocampus: α7nAChR ^+^ neurons↑, TNF-α ^+^ neurons↓, IL-1β ^+^ neurons↓
[143]	3 × Tg-AD mice	EA	GV20, GV24	1/20 Hz, 1 mA, 30 min, 5 days a week, 4 weeks	NORT: recognition index↑	ReHo in the amygdala, auditory cortex, DG, dorsal raphe nucleus, entorhinal cortex, hippocampus, somatosensory cortex, subiculum, substantia nigra, temporal cortex, and ventral tegmental area↑; postsynaptic sEPSC of hippocampus CA1↑; FC of the hippocampus with entorhinal cortex↑
[144]	D-galactose injection rats	MA	ST36	Twisting at 90–180°, 60–90 times/min; lifting and thrusting at 0.1–0.2 mm, 60–90 times/min; 30 min	——	Glycol metabolism in the pyriform cortex of the right limbic system, the olfactory cortex of the right temporal lobe, the right amygdaloid body, the right hippocampus, the pyriform cortex of the left limbic system, and the olfactory cortex of the left temporal lobe↑
[145]	MCAO rats	EA	GV20, GV24	1/20 Hz, 0.2 mA, 30 min, 14 days	MWM test: escape latency↓, platform cross number↑	FC of the left RSC with the left hippocampus, left RSC, right RSC, left CG, right CG, right tegmentum of midbrain and right visual cortex↑
[147]	AD patients (n = 14)	MA	LR3, LI4	3 min	–	ALFF in the left PoCG↑; FC of the right hippocampus with the left PrCG↑
[148]	AD patients (n = 14)	MA	LR3, LI4	3 min	–	FC of the MFG with the left hippocampus↑
[151]	AD patients (n = 14)	MA	LR3, LI4	3 min	–	FC of the bilateral CG with left PCu↓, FC of the right IPL, right MTG, with a cluster in left PCC↑
[152]	D-gal injection rats	MA	HT7	Twisted/rotated at 120–150 times per min, 3 min, 2 min break and repeated three times, 5 days a week, 6 weeks	Y-Maze test: total reaction time↓	Glucose metabolism in the hippocampus, orbital cortex, cerebellum, piriform cortex, RSC, sensory cortex, and olfactory cortex↑
[153]	MCI patients (n = 12)	MA	KI3	Twisted at 60°, 120 times/min, 2 min	–	Activities in bilateral anterior cingulate gyrus, left medial frontal gyrus, left cuneus, left middle frontal gyrus, left lingual gyrus, right middle frontal gyrus, bilateral inferior frontal gyrus, left SFG, right cuneus, right STG, left subcallosal gyrus, bilateral PCu, right medial frontal gyrus, right SFG, left CG, left PrCG, and right FG↑
[154]	MCI patients (n = 8), AD patients (n = 12)	MA	LR3, LI4	3 min	–	In the procession of acupuncture: activities in the bilateral CPL, bilateral MTG, bilateral FG, right PHG, left ITG, frontal lobe, bilateral IPL, right PoCG and occipital lobe↑, activities in the bilateral CPL, temporal lobe, bilateral SFG, right MFG, left PrCG, right PoCG, left PCL, left SPL, right lingual gyrus and limbic regions↓ of MCI patients; activities in the right CPL, bilateral frontal lobe, right IPL, right MOG↑, activities in the right STG, right MTG, bilateral MFG and left brain stem↓ of AD patients; In the second resting state after acupuncture: In MCI patients, activities in the bilateral CPL, bilateral FG, right MTG and right PHG, frontal lobe, right lentiform nucleus, left extra nuclear and right thalamus↑, activities in the bilateral CPL, bilateral MTG, left STG, right ITG and right FG, left SFG, left IFG, bilateral PrCG, right MFG, bilateral PoCG, left IPL, bilateral SPL, right angular and left SOG, left cuneus↓ of MCI patients; activities in the right CPL, left ITG, right MTG, bilateral SFG, left IFG, right MFG and bilateral PrCG, right MOG, bilateral SMG, right SPL↑, activities in the left CPL, bilateral PHG, right MFG, left lingual gyrus, right cingulate gyrus, left lentiform nucleus and right midbrain↓ of AD patients
[155]	MCI patients (n = 32)	MA	EX-HN1, EX-HN3, PC6, KI3, ST40, LR3	twirled at ± 60°, 120 times per min, 5 days a week, 4 weeks	MMSE↑, MoCA↑, digit-symbol task↑, digit-span task↑, word recall task↑	Insula, DLPFC, and hippocampus acted as central hubs. The insula received causal inflows from the thalamus, hippocampus, ACC, and primary somatosensory cortex. The hippocampus received causal inflows from the DLPFC, ACC, and mPFC. The DLPFC received causal inflows from the OFC, ACC, and primary motor cortex. The IPL received causal inflows from the DLPFC and culmen. The PCu received causal inflows from the FG, the thalamus received causal inflows from the caudate. The supplementary motor area received causal inflows from the insula. The somatosensory cortex received causal inflows from the MTG

↑, upregulated by intervention; ↓, downregulated by intervention

Aβ, amyloid-beta; MA, manual acupuncture; GV24, *Shenting*; GB13, *Benshen*; ChAT, choline acetyltransferase; AChE, acetylcholinesterase; SOD, superoxide dismutase; MDA, malondialdehyde; ROS, reactive oxygen species; EA, electroacupuncture; GV20, *Baihui*; BL23, *Shenshu*; MWM, Morris Water Maze; MCAO/R, middle cerebral artery occlusion-reperfusion; NF-κB, nuclear factor-kappa B; phosphorylated IκB, phosphorylated NF-κB inhibitor; BCCAO, bilateral common carotid artery occlusion; GV14, *Dazhui*; GV29, *Yintang*; GV26, *Shuigou*; JNK, c-Jun N-terminal kinase; MKK7, mitogen-activated protein kinase kinase 7; SP600125, JNK inhibitor; 2VO, bilateral common carotid artery occlusion; ST36, *Zusanli*; Trx-1, thioredoxin-1; TrxR-1, thioredoxin reductase-1; ASK1, apoptosis signal-regulating kinase 1; APP, β-amyloid precursor protein; BACE1, Beta-site APP cleaving enzyme1; PKA, protein kinase; OFT, Open Field Test; Akt, protein kinase B; GSK3β, Glycogen synthase kinase-3; CV17, *Danzhong*; CV12, *Zhongwan*; CV6, *Qihai*; SP10, *Xuehai*; DG, dentate gyrus; GSH-Px, glutathione peroxidase; HSP84, heat shock protein 84; HSP86, heat shock protein 86; GLUT1, glucose transporter type 1; GLUT3, glucose transporter type 3; AMPK, AMP-activated protein kinase; mTOR, mammalian/mechanistic target of rapamycin; PI3K, phosphatidylinositol 3-kinase; FL-APP, full-length APP; LC3B, microtubule-associated protein light chain 3 beta; SQSTM1, sequestosome 1; CTSD, cathepsin D; LAMP1, lysosomal-associated membrane protein 1; TFEB, transcription factor EB; RPS6, ribosomal protein S6; PFC, prefrontal cortex; MAPK, mitogen-activated protein kinases; CQ, chloroquine; CTFs, beta C-terminal fragments; GV16, *Fengfu*; ATP, adenosine triphosphate; iNOS, inducible nitric oxide synthase; TOMM40, Translocase of Outer Mitochondrial Membrane-40; TOMM17A, Translocase of Outer Mitochondrial Membrane-17A; COX, cyclooxygenase; LI/R, Limb ischemia/reperfusion; GB34, *Yanglingquan*; LR3, *Taichong*; 8-OH-dG, 8-Hydroxy-2'-deoxyguanosine; T-AOC, total antioxidant capacity; NOX2, nicotinamide adenine dinucleotide phosphate oxidases 2; LA, laser acupuncture; HT7, *Shenmen*; CAT, catalase; TBCA, NADPH oxidase activator; CBF, cerebral blood flow; Nrf2, Nuclear factor erythroid 2-related factor2; HO-1, heme oxygenase-1; NQO1, NAD(P)H: Quinone Oxidoreductase 1; Arg1, arginase-1; BVA, Bee Venom Acupuncture; TLR4, Toll-like Receptor 4; TNF-α, Tumour necrosis factor alpha; BDNF, brain-derived neurotrophic factor; ERK, extracellular signal-regulated kinase; IL-6, interleukin-6; MyD88, myeloid differentiation primary response 88; IL-1β, interleukin-1beta; IL-18, interleukin-18; NLRP3, nucleotide-binding oligomerization domain-like receptor family pyrin domain-containing-3; ASC, apoptosis-associated speck-like protein; GSDM-D, gasdermin-D; TXNIP, thioredoxin-interacting protein; α-BGT, α7nAChR antagonist α-bungarotoxin; α7nAChR, alpha7 nicotinic acetylcholine receptor; JAK2, Janus kinase 2; STAT3, signal transducer and activator of transcription 3; GFAP, Glial fibrillary acidic protein; P2X7R, P2X purinergic receptor 7, ligand-gated ion channel, 7; P2Y1R, P2Y purinergic receptor 1; MID, multi-infarction dementia; NSCs, neural stem cells; bFGF, basic fibroblast growth factor; EGF, epidermal growth factor; NeuN, neuronal nuclei; BrdU, bromodeoxyuridine; NORT, novel object recognition task; DCX, doublecortin; SGZ, subgranular zone; LV-CC, lateral ventricle-corpus callosum; NLRT, novel location recognition; MS/VDB, medial septum/vertical limb of the diagonal band of Broca; NAA, N-acetyl aspartate; Cr, creatine; Cho, choline; M1 mAChR, M1 muscarinic acetylcholine receptor; VAChT, vesicular acetylcholine transporter; BCAS, bilateral carotid artery stenosis; CC, corpus callosum; MBP, myelin basic protein; NG2, neural/glial antigen 2; CNPase, 2′,3′-cyclic nucleotide-3′-phosphodiesterase; PDGFRα, platelet-derived growth factor receptor alpha; Figf, C-Fos-Induced Growth Factor; Mdk, midkine; NT4/5, neurotrophin-4/5; TrkB, tropomysin related kinase B; CREB, cAMP Response Element-Binding Protein; ANA-12, TrkB antagonist; PSD-95, postsynaptic density protein-95; Cdc42, cell division cycle 42; Rac1, small GTPase Ras-related C3 botulinum toxin substrate 1; RhoA, Ras homolog gene family member A; EX-HN3, *Yintang*; NMDAR1, N-methyl-d-aspartate receptor 1; AMPAR, AMPA-type glutamate receptor; GABA_A_R, type A gamma-aminobutyric acid chloride channel; CaMKII, Calcium/calmodulin-dependent protein kinase II; fEPSP, field excitatory postsynaptic potential; cAMP, 3', 5' cyclic adenosine monophosphate; H89, N-(2-(4-bromocinnamylamino) ethyl)-5-isoquinolinesulfonamide; PP-DG, perforant pathway-dentate gyrus; CSF, cerebrospinal fluid; SYP, Synaptophysin; DOPAC, 3,4-dihydroxyphenylacetic acid; HVA, homovanillic acid; D1R, Dopamine D1 Receptor; D5R, Dopamine D5 Receptor; SCH23390, D1/D5 receptors antagonist; SKF38393, D1/D5 receptor agonist; NE, norepinephrine; β1-AR, beta 1-adrenergic receptor; RAM, Radial Arm Maze; DBH, Dopamine β-Hydroxylase; Glu, Glutamate; NMDAR2A, N-methyl-d-aspartate receptor 2A; NMDAR2B, N-methyl-d-aspartate receptor 2B; SCO, scopolamine; TE4, *Yangchi*; PAT, passive avoidance test; CHT1, choline transporter; POCD: Postoperative Cognitive Dysfunction; PC6: *Neiguan*; sEPSCs, spontaneous excitatory postsynaptic currents; FC: functional connectivity; RSC: retrosplenial cortex; CG: cingulate gyrus; LI4:*Hegu*; AD: Alzheimer’s disease; ALFF, amplitude of low-frequency fluctuation; PoCG, postcentral gyrus; PrCG, precentral gyrus; FG, fusiform gyrus; MFG, middle frontal lobe; PCu, precuneus; IPL, inferior parietal lobule; MTG, middle temporal gyrus; PCC, posterior cingulate cortex; MCI, mild cognitive impairment; KI3, *Taixi*; SFG, superior frontal gyrus; STG, superior temporal gyrus; CPL, cerebellum posterior lobe; PHG, parahippocampus; ITG, inferior temporal gyrus; PCL, paracentral lobule; SPL, superior parietal lobule; MOG, middle occipital lobe; SOG, superior occipital lobe; EX-HN1, *Sishencong*; ST40, *Fenglong*; MMSE, Mini-Mental State Examination; MoCA, Montreal Cognitive Assessment; DLPFC, dorsolateral prefrontal cortex; ACC, anterior cingulate cortex; mPFC, medial prefrontal cortex; OFC, orbitofrontal cortex

### Mechanisms of acupoint stimulation in the treatment of cognitive impairment

The pathological process of dementia has not been fully elucidated. However, a variety of pathophysiological processes may potentially contribute to dementia, such as age, genetic predisposition, cardiovascular disease, diabetes, psychiatric disorders, traumatic brain injury, and obesity [26, 27]. Most dementias are associated with a loss of hippocampal neurons. Numerous basic studies have shown that acupoint stimulation mainly in the form of MA and EA improves a variety of primary and secondary cognitive impairments, including those caused by AD, VD, Parkinson’s disease (PD), drugs, sepsis, and radiation brain injury, mainly using the measure of improved spatial learning memory in animal models. This study describes how acupoint stimulation helps protect, regenerate, and plasticize hippocampus neurons and restores proper function to neuronal circuits, as shown in Table1.

### Neuroprotective mechanism of acupoint stimulation in the treatment of cognitive impairment

#### Anti-apoptosis

Apoptosis of neurons in the hippocampus or cortex is an important event in the progression of AD and VD diseases [28, 29]. Apoptotic pathways mainly include the intracellular mitochondrial and the cell surface death receptor pathway [30]. Damage signals by toxic proteins, oxidative stress, and inflammation generated during the pathology of cognitive impairment may indirectly induce these two apoptotic cascade pathways, causing neuronal apoptosis and ultimately cognitive impairment. Acupuncture promotes neuronal survival mainly by regulating factors involved in the intracellular apoptotic pathways. Acupuncture at *Benshen* (GB13) and GV24 increased Bcl-2 and decreased Bax, CYC, caspase-3, and caspase-9 in the hippocampi of AD rats [31]. Additionally, 20 Hz EA at GV20 and BL23 upregulated Bcl-2 and downregulated Bax [32], suggesting that both MA and EA could reduce hippocampal neuronal apoptosis in AD rats. Moreover, similar results were obtained in VD rats and POCD rats [33–35]. Furthermore, in rats with hypergravity-induced cognitive impairment, 2/15 Hz EA pretreatment at GV20 attenuated caspase-3 activity and neuronal apoptosis in the CA1 region [36]. Suspension moxibustion at GV20 and BL23 reduced apoptosis in rat hippocampal neurons exposed to amyloid β-peptide 1–42 (Aβ_1–42_). In particular, administering moxibustion before Aβ_1-42_ exposure improved the protection of neural structures and decreased apoptosis following Aβ_1-42_ exposure [37]. These results indicate that both electroacupuncture and moxibustion may prevent cognitive impairment by blocking neuronal apoptosis.

It has been shown that nuclear factor-κB (NF-κB) plays a considerable role in preventing neuronal death. It was found that 1–20 Hz EA at GV20 and GV24 inhibited the activation of NF-κB signaling induced by cerebral ischemia–reperfusion (I/R) and downregulated the expression of two key target genes related to apoptosis, Bax and Fas, downstream of the NF-κB pathway [38]. Moreover, it has been hypothesized that EA’s ability to suppress both endogenous and exogenous apoptotic pathways contributes to its beneficial effect on cognitive impairment in VD rats. Activation of p53, a downstream target gene of NF-κB, can inhibit the expression of anti-apoptotic genes and activate the expression of pro-apoptotic genes that are involved in mitochondria-dependent apoptotic pathways [39]. It was found that p53 expression in the hippocampus of VD rats was positively correlated with Noxa expression. Additionally, some studies demonstrated that EA may inhibit apoptosis in VD rats and may be correlated with suppressing the expression of p53 and Noxa in the CA1 region after exposure to 4 Hz EA at GV20, GV14, and BL23 [40]. It was reported that 1 Hz EA at GV20 and *Yintang* (GV29), and puncture at GV26 decreased the expression of mitogen-activated protein kinase kinase 7 (MKK7), p–c-Jun, p-MKK7, and c-Jun, thereby inhibiting the c-Jun N-terminal kinase (JNK) signaling pathway and regulating apoptotic signaling and reversing cognitive impairment in amyloid precursor protein (APP)/presenilin 1 (PS1) mice [41, 42]. In chronic cerebral hypoperfusion (CCH), there is an accumulation of cerebral reactive oxygen species (ROS) and oxidation of thioredoxin-1 (Trx-1). This leads to the activation of the signal-regulated kinase 1 (ASK1)-JNK/p38 pathway and apoptosis [43]. Acupuncture at GV20 and ST36 upregulated Trx-1 and thioredoxin reductase-1 (TrxR-1) expression and increased TrxR-1 activity while inhibiting ASK1-JNK/p38 pathway activation, while these effects were blocked by Trx-1 siRNA [44]. Thus, it is hypothesized that acupuncture can enhance Trx-1 and TrxR-1 while suppressing the ASK1-JNK/p38 pathway to alleviate VD-associated cognitive impairment. In this view, NF-κB/p53 and ASK1-JNK/P38 signaling cascades may have a significant role in the process through which acupuncture promotes neuronal survival by modulating apoptosis-related variables.

#### Scavenging toxic proteins 

Amyloid β-peptides (Aβ) aggregation between neurons in regions including the hippocampus and cortex results in senile plaques, and increased levels of phosphorylated Tau protein (p-Tau) that further affect the stability of microtubules lead to neurofibrillary tangles, both of which cause neuronal death and cognitive impairment [3]. Acupuncture may improve cognitive impairment in AD rats by inhibiting the production of toxic proteins and promoting their clearance. First, acupuncture can inhibit the production of Aβ. Beta site amyloid precursor protein-cleaving enzyme 1 (BACE1) is a key protein involved in the production of the Aβ peptide, cleaving the peptide from the APP. It was found that 2 Hz EA at GV20 and GV29 and puncture at GV26 reduced the co-expression and deposition of BACE1 and APP in the hippocampus of APP/PS1 mice, thereby improving memory and learning ability [45]. Several studies have reported that acupuncture could also decrease the level of phosphorylation at specific Tau protein phosphorylation sites. Additionally, many serine phosphorylation sites on the Tau protein were found to be phosphorylated in AD mice, including Ser199, Ser202, Ser396, and Ser404 [46–48]. The mechanism underlying the improvement of cognitive function by EA at GV24 and GB13 may be related to the reduction of Aβ, p-Tau (Ser396), and p-Tau (Ser404) in the hippocampi of AD rats [49]. Additionally, 2 Hz EA at GV20 and BL23 caused a decrease in hippocampal Aβ and p-Tau (Ser404) [50]. EA with the same frequency at GV20, GV29, and GV26 enhanced glucose metabolism in APP/PS1 mice, activated protein kinase B (AKT), decreased glycogen synthase kinase-3beta (GSK-3β) activity, promoted GSK3β (Ser9) phosphorylation, and eventually inhibited hippocampal phosphorylation of Tau (Ser199 and Ser202) [51, 52].

Both HSP86 and HSP84 protect neuronal function by degrading misfolded proteins and preventing the formation and aggregation of Aβ and Tau [53]. It has been reported that acupuncture might promote correct folding in potentially toxic proteins; for example, acupuncture at *Danzhong* (CV17), *Zhongwan* (CV12), *Qihai* (CV6), *Xuhai* (SP10), and ST36 improved cognitive function and increased neuronal numbers in senescence-accelerated mouse prone 8 (SAMP8) mice, accompanied by increased expression of HSP84 and HSP86 [53].

By triggering autophagy, acupuncture can help remove misfolded and aggregated proteins. It was discovered that 1/20 Hz EA at GV20 caused AMP-activated protein kinase and AKT to become phosphorylated while inhibiting the phosphorylation of the mammalian rapamycin target (mTOR) [54]. Inactivating mTOR activated the autophagic pathways to manage the Aβ accumulation in the cortex and hippocampus regions. EA at the same frequency reversed the decrease in the microtubule-associated protein, 1A/1B-light chain 3 II (LC3II)/microtubule-associated protein, 1A/1B-light chain 3 I ratio, and Beclin-1 levels in the hippocampus after Aβ_1-40_ injection. It also induced the expression of autophagic precursors and larger autophagosomes, decreasing the Aβ levels. The co-localization of Aβ and LC3II suggests that EA-induced autophagy in Aβ_1-40_ injected rats eliminates the Aβ aggregations [55]. In contrast, Zheng et al. found that in the prefrontal cortices and hippocampi of 5xFAD mice, LC3B, sequestosome 1 (SQSTM1), and lysosome-associated membrane protein 1 (LAMP1) aggregated around or co-localized with APP/Aβ plaques, which may indicate defects in autophagic cargo recognition, trafficking to lysosomes, and lysosomal activity. The insoluble LC3B-II and SQSTM1 were decreased at GV24 and GB13 by 2 Hz EA, and the reduced LC3B^+^ cathepsin D (CTSD) area was restored in the prefrontal cortex and hippocampus. Increased CTSD and LAMP1 levels were found in both the precursor and mature forms, indicating that EA may not have an impact on the induction of autophagy but primarily promotes lysosomal biogenesis and destroys insoluble SQSTM1 and APP/Aβ. As a result of inhibiting AKT, mTOR complex 1, and mitogen-activated  protein kinases 1, EA may also activate transcription factor EB (TFEB). Activated TFEB transcription increases autophagy-lysosomal degradation of APP, the production of APP C-terminal fragments, and Aβ [56]. Additionally, it was discovered that acupuncture at the GV20, GV14, GV26, and *Fengfu* (GV16) points inhibited the buildup of Aβ in the mitochondria as well as the expression of outer mitochondrial membrane 40, which is involved in encouraging Aβ influx into the mitochondria, thereby reducing mitochondrial dysfunction [57, 58].

In view, acupuncture prevents the synthesis of Aβ and the phosphorylation of Tau protein. Additionally, it encourages proper protein folding and controls the autophagic degradation of toxic proteins, which may help to prevent neuronal damage.

#### Anti-oxidation

The imbalance between oxidative and antioxidant systems leads to damage to hippocampal neurons. Acupuncture at GV20, GV14, GV26, and GV16 inhibited the production of two pro-oxidative stress factors (i) nitric oxide and (ii) inducible nitric oxide synthase (iNOS) in VD rats [58], while the levels of ROS and malondialdehyde (MDA) in the hippocampus were decreased by pretreatment with 2/15 Hz EA at GV20, *Yanglingquan* (GB34), LR3, ST36, and SP10 for 14 days before limb ischemia–reperfusion (LI/R) [59]. In AD rats, EA at the same frequency at GV20 and Yongquan (KI1) had comparable effects [60]. On the other hand, acupuncture at CV17, CV12, CV6, SP10, and ST36 was related to reductions in superoxide anions and carbon bases and increased levels of plasma glutathione peroxidase (GSH-Px) and superoxide dismutase (SOD) in the hippocampus. This also improved cognitive function in SAMP8 mice [53]. 2/15 Hz EA pretreatment at GV20, GB34, LR3, ST36, and SP10 also increased SOD activity in the CA1 region of VD rats, while LA at HT7 enhanced both SOD and catalase (CAT) activities in the hippocampi of AD rats [59, 61]. Acupuncture, meanwhile, demonstrated comparable effects in models of cognitive impairment brought on by various pathological conditions, including acute myocardial infarction/reperfusion, Parkinson's disease, surgery, and lipopolysaccharide administration [62–65].

As a result, increased SOD brought on by stimulation of acupoints may turn superoxide free radicals into hydrogen peroxide. GSH-Px and CAT can stop hydrogen peroxide from entering cells to produce hydroxyl free radicals, thereby reducing oxidative stress [66].

Additionally, it has been demonstrated that acupuncture inhibits nicotinamide adenine dinucleotide phosphate oxidases(NOXs), preventing the production of ROS. In the ischemic brain, NOX2 (gp91phox) and NOX4 are significant sources of ROS [67, 68]. Treatment with 2/15 Hz EA at GV20 and KI1 reduced abnormally elevated hippocampal NOX2 levels and Aβ_1-42_-induced hippocampal neuronal damage in AD rats [60]. Activation of NOX2 is dependent on the phosphorylation of the cytoplasmic protein p47phox [69]. Acupuncture at GV20 and ST36 suppressed bilateral common carotid artery occlusion (2VO)-induced elevation of gp91phox and p47phox in cognition-impaired rats [70]. Meanwhile, acupuncture increased the expression of nuclear factor erythroid2-related factor2 (Nrf2) and heme oxygenase 1 (HO-1) in VD rats [71]. On the one hand, Nrf2 is activated by binding to the antioxidant response element, which stimulates the transcription of antioxidant proteins such as HO-1 and participates in the synthesis of glutathione [72], while, on the other hand, there is a negative feedback regulatory loop between NOX4 and Nrf2. Under steady-state conditions, NOX4 generates superoxide and hydrogen peroxide to activate Nrf2 [73], and the activated Nrf2 inhibits NOX4 transcription to reduce ROS production [72]. Acupuncture may initiate this complex signaling pathway.

By decreasing ROS, MDA, and other oxides and raising SOD, GSH-Px, CAT, and other antioxidants in the hippocampal neurons, acupuncture may be able to restore the oxidation-antioxidant balance of the cognition-impaired brain. This regulation process may involve NOXs and Nrf2-related signaling pathways.

#### Anti-neuroinflammation

Cognitive functions have been found to suffer from chronic neuroinflammation [74]. A key component of neuroinflammation in the central nervous system is microglial activation. Excessive activation of M1-type microglia causes the release of large quantities of inflammatory mediators and the induction of neuronal apoptosis [75]. In the parietal association cortex and entorhinal cortex of mice with mild AD, EA (1/20 Hz) at GV20 and GV24 decreased the colocalization of iNOS/interleukin-1beta (IL-1β), and Iba1 (M1 microglial markers), and increased the colocalization of CD206/arginase-1 and Iba1 (M2 microglial markers), indicating that EA regulates microglial polarization [76]. Acupuncture at GV29 and Yingxiang (LI20) and 2 Hz EA at GV24 and GB13 prevented AD mice's hippocampi from becoming activated by microglia [56, 71]. According to another study, pretreatment with 2/15 Hz EA before LI/R decreased microglial activation in the CA1 region [36]. Additionally, in AD and VD rats, MA and EA reduced the production of pro-inflammatory cytokines like tumor necrosis factor-alpha (TNF-α), interleukin-6 (IL-6), and IL-1β [77, 78].

Through their cell surface receptor complexes, microglia interact with Aβ protofibrils, which in turn causes phagocytosis of Aβ and neuroinflammation. Toll-like receptor 2, Toll-like receptor 4, and their co-receptor CD14 are the microglial receptor complexes that recognize Aβ protofibrils [79]. Bee venom acupuncture at the ST36 acupoint enhanced cognitive performance in VD Mongolian gerbils, attenuated microglial activation in the hippocampus, and suppressed the expression of TLR4, CD14, and TNF-α [80]. Acupuncture at GV20 and ST36 suppressed TLR4 expression in the hippocampal microglia of VD rats, accompanied by reduced activation of miR-93 and myeloid differentiation primary response 88 (MyD88)/NF-κB signaling, suggesting that acupuncture may attenuate inflammation-related cognitive impairment by inhibiting miR-93-mediated TLR4/MyD88/NF-κB signaling in VD rats [81]. Other studies using EA reported similar results, implying that TLR4/MyD88/NF-κB signaling may have mediated EA’s effect in rats with VD and hepatic encephalopathy [82, 83].

Microglia promote the cleavage of pro-caspase-1 to active caspase-1 by activating NLR family pyrin domain-containing 3 (NLRP3), causing the release of pro-inflammatory cytokines such as IL-1β and TNF-α [84]. In SAMP8 mice, 10 Hz EA at GV20 and ST36 was more effective than 2 Hz EA in reducing the number of TUNEL^+^ cells and serum IL-1β and IL-6 levels in the CA1 region, which may be associated with the downregulation of hippocampal NLRP3/caspase-1 pathway-related proteins [85]. Acupuncture at the same acupoints restored the 2VO-induced elevation of hippocampus thioredoxin-interacting protein (TXNIP), NLRP3, caspase-1, and IL-1β, indicating that acupuncture may perform neuroprotective effects in VD rats by decreasing TXNIP-related oxidative stress and inflammation [86]. Another research obtained similar results and the NLRP3 activator abolished the anti-inflammation effect on the cognitive function of EA treatment [87]. Moreover, acupuncture repressed microglial activation and TNF-α, IL-6, and IL-1β levels, which may be related to the up-regulation of the α7-nicotinic acetylcholine receptor (α7nAChR) and its downstream pathway including high mobility group box 1 (HMGB1)/NF-κB or Janus kinase 2 (JAK2)/signal transducer and activator of transcription 3 (STAT3) pathway by acupuncture [88–92].

Some studies have revealed that 2/20 Hz EA at GV20 and GV24 reduced astrocyte proliferation and microglial/macrophage activation, decreased IL-1β secretion, and promoted IL-10 release, accompanied by a decrease in purinergic P2X receptor 7 (P2X7R)^+^ED1^+^, P2X7R^+^GFAP^+^, purinergic P2Y receptor 1 (P2Y1R)^+^ED1^+^, and P2Y1R^+^GFAP^+^ cells in the CA1 region of the peri-infarct hippocampus and sensorimotor cortex. These findings suggest that EA exerts anti-inflammatory effects by inhibiting astrocyte and microglial/macrophage P2X7R and P2Y1R-mediated neuroinflammation after middle cerebral artery occlusion/reperfusion injury and improves motor and memory functions [93].

The above evidence suggests that acupuncture may protect hippocampal neurons from neuroinflammatory damage by regulating TLR4/MyD88/NF-κB, HMGB1/NF-κB, JAK2-STAT3, and P2 purinergic receptor signaling pathways.

### Acupoint stimulation modulates neural regeneration and neuroplasticity in the treatment of cognitive impairment

#### Promoting neural regeneration

Multiple studies have reported that acupuncture may stimulate neuronal regeneration. Acupuncture at ST36 has been demonstrated to enhance the level of pyramidal neurons in the CA1 region of VD rats [94]. Additional studies suggested that acupuncture at CV17, CV12, CV6, SP10, and ST36 elevated the number of CA3 and dentate gyrus (DG) neurons in the hippocampus [95]. It was reported that acupuncture at CV17, CV12, CV6, ST36, and SP10 increased the number of NeuN^+^/BrdU^+^ cells in the DG after neural stem cell (NSC) transplantation in SAMP8 mice, suggesting that acupuncture promotes NSC proliferation [96]. In rats with cognitive dysfunction after brain X-ray irradiation, 2/15 Hz EA at GV20, ST36 upregulated DCX^+^ neurons in the subgranular zone of the hippocampus [97]. Another study reported that acupuncture reversed declining cell proliferation in the DG and showed a stream-like distribution along the dorsum of the sulcus that extended from the left ventricle to the corpus callosum (CC) [98].

By regulating neurotrophic and nerve growth factors, acupuncture may improve neural regeneration. Treatment of AD mice with 2/15 Hz EA at GV20 enhanced the expression of brain-derived neurotrophic factor (BDNF) in the hippocampus and cortex [99]. Additionally, the basic fibroblast growth factor (bFGF) and epidermal growth factor (EGF) are two key mitogens involved in the proliferation of NSCs [100, 101]. Acupuncture upregulated the expression of BDNF, bFGF, and EGF in the hippocampus of SAMP8 mice after NSC transplantation [96]. An interesting finding was that the number of M1-type muscarinic acetylcholine receptors (M1 mAChR) in the medial septum (MS)/vertical limb of the diagonal band of Broca (VDB)-DG region increased after 2/20 Hz EA treatment at GV20 and GV24, with an increase in DCX^+^ cells and Neuro-D1^+^ cells, however, the above effects of EA vanished when hM4Di Designer Receptors Exclusively Activated by Designer Drugs (DREADDs) were used to block cholinergic circuits in this area. This evidence revealed that M1 mAChR may also facilitate EA-induced neuronal regeneration in the MS/VDB-DG region, although further research is required to confirm this hypothesis [102].

Additionally, acupuncture may alleviate damage to the neuronal myelin sheath and stimulate the regeneration of oligodendrocytes, which will lead to neural regeneration. For instance, treatment with 2 Hz EA at GV20 and GV14 reduced the number of new oligodendrocyte precursor cells (OPCs) in the CC and increased the number of newly differentiated oligodendrocytes (OLs). In addition, EA also increased the levels of neurotrophin 4/5(NT4/5)-tropomysin related kinase B (TrkB) /cAMP Response Element-Binding Protein (CREB) in the OLs and OPCs of the CC, suggesting that the effects of EA on oligodendrocyte regeneration were related to NT4/5-TrkB signal transduction [103].

The aforementioned research suggests that acupuncture promotes neural regeneration by activating factors including BDNF, bFGF, and EGF, and by repairing injuries to neuronal myelin sheaths, hence reducing cognitive impairment.

#### Modulating synaptic plasticity

Synaptic plasticity plays a key role in learning and memory and includes both structural and functional plasticity [104]. Reduced hippocampal synaptic plasticity in AD and VD patients results in impaired spatial learning and memory [105, 106]. Structural plasticity depends on the synaptic ultrastructure, such as the synaptic curvature, synaptic cleft width, and postsynaptic density. Acupuncture at CV17, CV12, CV6, SP10, and ST36 increased the expression of synaptophysin (SYN, a presynaptic vesicle marker) in SAMP8 mice after receiving NSC transplantation [107]. In AD mice, treatment with 2 Hz EA increased SYN and the postsynaptic marker postsynaptic density protein 95 (PSD-95) and decreased synaptic ultrastructural deterioration [108–110].

Various studies have shown that BDNF/TrkB and its downstream signaling pathways may mediate the regulation of synaptic plasticity by acupuncture. It was reported that EA increased the expression of SYP, PSD-95, BDNF, and TrkB in the hippocampus [111, 112]. Furthermore, the binding of BDNF to TrkB activates the downstream phosphatidylinositol-3-kinase (PI3K) /AKT pathway. Acupuncture at GV29 and LI20 increased the expression of PSD-95, SYN, and growth-associated protein 43 in SAMP8 mice, and activated the PI3K/AKT signaling pathway and the phosphorylation of GSK-3β [55]. GSK-3β, in turn, downregulates BDNF and destroys synaptic plasticity by impairing CREB protein transcriptional activity. Potentially, 2/30/50 Hz EA can downregulate GSK-3β, leading to an increase in synaptic bending, a decrease in synaptic cleft width, and an increase in postsynaptic density. In resting settings, GSK-3β is highly active and can be triggered by phosphorylation at Tyr216 and inhibited by phosphorylation at Ser9 [113]. After EA treatment, pSer9-GSK-3β expression was found to be significantly reduced, while pTyr216-GSK-3β expression was found to be significantly increased; furthermore, EA with high frequency (50 Hz) was more effective than EA with low frequency (2 Hz) or medium frequency (30 Hz) [114]. PI3K/AKT, as molecules upstream of GSK-3β, can phosphorylate and inhibit GSK-3β. Consequently, it is hypothesized that the BDNF/TrkB-PI3K/AKT/GSK-3β signaling pathway may facilitate acupuncture’s modulation of synaptic structural plasticity in the hippocampus [115].

Rho GTPases and the Notch signaling pathway may also play roles in the regulation of synaptic structural plasticity by acupuncture. According to Lin et al., 20 Hz EA at GV20 and GV24 increased hippocampal dendritic spine densities in I/R-injured rats. This effect may have been caused by upregulating cell division cycle 42 and Ras-related C3 botulinum toxin substrate 1 and downregulating Ras homolog gene family member A, which would have regulated the F-actin cytoskeleton and stimulated the growth of local dendritic spines [116]. Guo et al. found that the levels of Notch1 and Hes1 were abnormally elevated in the hippocampus after Aβ injection, whereas 20 Hz EA at GV20 and BL23 downregulated hippocampal Notch1 and Hes1 expression and promoted synapsin-1 and SYN expression, suggesting that EA partially improves learning memory by downregulating the abnormally elevated Notch signaling pathway in AD rats [32].

There are two main forms of synaptic plasticity, namely, long-term potentiation (LTP) and long-term depression. LTP is considered to be the molecular basis of memory formation [117]. Early-LTP induction requires activation of the postsynaptic NMDAR and Ca^2+^ flux into the activated NMDAR channel, which results in the activation of calcium/calmodulin-dependent protein kinase II (CaMKII) and a rapid increase in the number of α-amino-3-hydroxy-5-methyl-4-isoxazolepropionic acid receptors (AMPAR) at the synapse [118]. Late-LTP involves the activation of 3′, 5′ cyclic adenosine monophosphate (cAMP) by activated CaMKII, regulating the protein kinase A (PKA)/CREB pathway and increasing the number of AMPAR [119, 120]. EA increased the levels of CaMKII in the hippocampus [121, 122] while acupuncture at ST36 alleviated disordered LTP in the perforant pathway-dentate gyrus (PP-DG), and counteracted decreases in cAMP, PKA, and p-CREB in cerebral multi-infarction rats. Increased CREB phosphorylation and the improvement in cognitive function were both blocked by the PKA inhibitor N- [2-(p-bromocinnamyl amino) ethyl] -5-isoquinoline-sulfonamide [123]. Zheng et al. obtained similar results, observing enhanced LTP in Schaffer collaterals [124] and suggesting that the cAMP/PKA/CREB pathway may be involved in LTP induction by acupoint stimulation.

In AD patients, elevated levels of Orexin A in the cerebrospinal fluid were related to elevated levels of phosphorylated Tau and Aβ in the cerebrospinal fluid, as well as diminished cognitive scores [125, 126]. The latest study found that 10 Hz EA alleviated learning and memory impairment in SAMP8 mice, reduced the level of Orexin A in the cerebrospinal fluid, improved the synaptic structure and synaptic transmission of the hippocampus, increased the level of glutamate, SYP, PSD-95, cAMP, pPKA/PKA, and pCREB/CREB protein levels. Orexin A-RNAi adenovirus was utilized to silence the orexin gene, simulating the abovementioned consequences of EA. It is hypothesized that 10 Hz EA therapy alters glutamatergic synaptic plasticity mediated by cAMP/PKA/CREB by decreasing cerebrospinal fluid Orexin A levels [127]. Dopamine (DA) and norepinephrine (NE) and their receptors may also play important roles in synaptic plasticity. Acupuncture at ST36 and GV20 alleviated LTP injury in the PP-DG of VD rats and promoted the release of DA in the hippocampus while reversing the decreases in the DA receptors D1R and D5R in the DG region. It was also reported that the level of NE in the hippocampus and the numbers of β1-adrenergic receptors (β1-AR) in the DG region were increased by acupuncture. The protective effect of acupuncture on LTP was eliminated by blocking the D1/D5 receptor and use of a β1-AR antagonist, suggesting that the mechanism by which acupuncture maintains the LTP in VD rats may be related to increased levels of DA and NE and the activation of the D1/D5 receptor and β1-AR [128, 129]. They also observed that acupuncture promoted the expression and activity of dopamine β-hydroxylase in the cerebrospinal fluid of CCH rats [130], suggesting that acupuncture may also induce and maintain LTP in the hippocampus by regulating DA to NE conversion. Studies have demonstrated that DA acting on D1/D5 receptors increases cAMP production and improves NMDA channel function [131]. Hippocampal β-AR activation activates PKA by increasing intracellular cAMP production, thereby reducing the threshold for LTP induction [132]. However, it has to be determined whether these variations influence synaptic plasticity by modulating the classical LTP induction pathway. It has been observed that electrical stimulation at EX-HN3 and GV20 at 2 Hz increased the expression of NMDAR1, AMPAR, and -aminobutyric acid type A receptor [121]. Further research is required to determine whether the underlined result is related to EA’s role in the induction of LTP. Other studies demonstrated that middle cerebral artery occlusion (MCAO) elevates the concentrations of Ca^2+^, Glu, and NMDA2B in hippocampus cells and that excessive Glu continually activates postsynaptic NMDAR, leading to excessive Ca^2+^ influx and poor cognitive function. These abnormalities were eradicated with the use of 1/20 Hz EA at GV20 and GV24. Meanwhile, EA reversed the decline in NMDAR2A caused by MCAO. NMDAR2A contributes to neuronal regeneration, while NMDAR2B mediates oxidative stress-induced neuronal apoptosis. These results suggest that EA may reduce Ca^2+^ influx by inhibiting glutamate neurotoxicity and downregulating NMDAR2B expression [133].

The degeneration of cholinergic innervation is one of the causes of decreased neuroplasticity and memory decline. Acupuncture at GV24 and GB13 can enhance the learning and memory of AD model rats, and the mechanism may be related to the modulation of the activities of choline acetyltransferase (ChAT) and acetylcholinesterase (AChE) [31]. Similar effects were observed in rats treated with acupuncture for lipopolysaccharide administration [64], long-term corticosterone administration [134], and PD-induced cognitive impairment [62]. ChAT and AChE are the enzymes responsible for synthesizing and hydrolyzing acetylcholine (ACh), and their levels regulate ACh metabolism. Lee et al. reported that acupuncture at GV20 improved cognitive dysfunction induced by scopolamine, alleviated the decline in hippocampal ChAT levels, and restored the expression of the hippocampal choline transporter 1 and vesicular acetylcholine transporter [135], suggesting that acupuncture may regulate ACh metabolism, and may contribute to the circulation of choline from the synaptic cleft back to the presynaptic terminal, and improve the efficiency of vesicle filling. Li et al. reported that 2/20 Hz EA at GV20 and GV24 improved pattern separation disorders in 5xFAD mice, up-regulated ChAT and vesicular acetylcholine transporter, and down-regulated AChE in the MS/VDB-DG region, while the use of hM4Di DREADDs reversed the above effects of EA, suggesting that EA may improve early pattern separation by activating cholinergic system in MS/VDB-DG [102]. According to the reported study of Liu et al., 2/100 Hz EA at GV20, PC6, and LI4 could enhance learning and memory in rats with postoperative cognitive impairment, as well as upregulate α7nAChR^+^ neurons in the hippocampus regions [136]. Treatment with 2 Hz EA at GV20 inhibited the lipopolysaccharide-induced decrease in α7nAChR activity [64]. Cholinergic signaling can enhance hippocampal function, and the α7nAChR contributes to LTP induction in the hippocampus due to its high Ca^2+^ permeability [137, 138]. Therefore, it is speculated that acupuncture may activate the α7nAChR to protect synaptic plasticity by regulating the hippocampal cholinergic pathway.

### Neural circuit mechanism of acupoint stimulation in the treatment of cognitive impairment

The Papez circuit in the limbic system is involved in spatial learning and episodic memory [139]. The Papez circuit is composed of the medial temporal lobe (MTL, consisting of the entorhinal cortex, hippocampus, parahippocampal gyrus, and amygdala), mammillary bodies, anterior thalamic nuclei, and the cingulate cortex, which are interconnected by white matter tracts consisting of the fornix, papillary thalamic tract, and cingulate tract [140]. Both AD and VD patients may experience disruptions in the Papez circuit [141, 142]. Several lines of evidence demonstrate that acupuncture recovers the activity and functional connectivity (FC) of brain areas connected with this circuit. Lin et al. found that 1/20 Hz EA at GV20 and GV24 significantly increased FC between the hippocampus and entorhinal cortex, and this was also demonstrated by diffusion tensor imaging [143]. A PET study showed that acupuncture at ST36 mainly activated regions in the bilateral limbic system (piriform cortex), bilateral temporal lobe (olfactory cortex), right amygdala, and right hippocampus [144]. In MCAO rats, 1/20 Hz EA at GV20 and GV24 enhanced the FC between the retrosplenial cortex (RSC) and the hippocampus, cingulate gyrus (CG), and midbrain [145]. The RSC plays a significant role in learning and memory because it is more closely connected to the anterior thalamic nuclei within the cingulate cortex and receives projections from all of the anterior thalamic nuclei [146]. Zheng et al. found that acupuncture at LR3 and LI4 upregulated the amplitude of low-frequency fluctuations (ALFF) in the right superior frontal gyrus (SFG) and downregulated ALFF in the left posterior central gyrus, inferior cingulate cortex, right middle cingulate cortex, right inferior frontal gyrus (IFG), right hippocampus, and right inferior temporal gyrus in AD patients. Acupuncture also improved the FC between the hippocampus and the anterior central gyrus [147] as well as between the frontal and temporal lobes and the hippocampus [148].

The default mode network (DMN) is primarily involved in the formation of autobiographical memory and spans different cortical regions, including the precuneus (PCu), posterior cingulate cortex (PCC), inferior parietal lobe (IPL), and temporal and medial prefrontal cortex [149], which are heavily projected to the MTL [150]. These regions play different roles in resting-state brain activity, with the left middle temporal gyrus (MTG) providing information, the IPL spatial attention, and the PCC information integration [151]. Acupuncture can also regulate DMN activity and FC, especially with the frontal and temporal lobes, and can regulate FC within the DMN as well as FC between DMN and other cognition-related brain regions. Using PET imaging, Lai et al. revealed that acupuncture at HT7 enhanced glucose metabolism in the hippocampus, thalamus, hypothalamus, frontal lobe, and temporal lobe of AD rats [152]. Using fMRI technology, Chen et al. found that acupuncture at KI3 could activate the CG, frontal lobe, precuneus, and other brain regions in elderly MCI patients and healthy elderly volunteers [153]. Acupuncture at LR3 and LI4 reduced the elevated left SFG and right IFG activity in MCI patients and activated the previously inhibited CG and fusiform gyrus, suggesting that acupuncture can bidirectionally regulate brain activity in MCI patients [154]. Meanwhile, acupuncture may increase the FC among the left PCC, right MTG, and right IPL while decreasing the FC between the bilateral CG and left PCu in the DMN of AD patients. Tan et al. found that after acupuncture, the FC between the hippocampus and the insula, the dorsolateral prefrontal cortex, and PCu, and the insula and dorsolateral prefrontal cortex was increased [155].

Acupoint stimulation may improve cognitive impairment in MCI and AD patients and animal models by modulating the activity and FC of related brain regions within the Papez circuit and DMN. Furthermore, deep acupuncture at KI3 causes more significant alterations in brain function than superficial acupuncture, though additional research is required to evaluate whether this remains true for other acupuncture locations. The choice of acupoints and their associated parameters varies throughout studies, as does their standardization. All of these factors must be taken into consideration to produce results that can be used universally.

## Discussion and conclusion

Treatment of cognitive impairment using acupoint stimulation, especially MA and EA, has promising results. The acupoints chosen for the preliminary research were mostly found in the upper and lower limbs and the head and neck, such as GV20, ST36, GV24, GV14, and HT7. Among these, GV20 was the most commonly used. Data mining showed that the use of GV20 or the combination of other acupoints centered on GV20 might have a better therapeutic effect on AD and VD in clinical studies [156, 157], and basic studies showed a high degree of similarity. The combination of GV20 and ST36 has been the subject of the most research and was found to be effective in every pathological condition targeted by acupoint stimulation for cognitive impairment, particularly in the treatment of neuroinflammation. The parameters of EA are also crucial in determining its efficacy, and low and medium frequency EA, primarily 2 Hz and 2/15 Hz, has been the subject of the most research and yielded great results. However, some studies showed that while 2 Hz EA improved learning and memory in rats, 50 Hz was more effective [158], and another study reported that 30/100 Hz EA also improved cognitive function [77]. Therefore, to standardize treatment settings, additional research is required to examine the efficacy of using various EA frequencies and the efficacy of variable frequency EA versus single frequency EA.

**Fig. 2:**
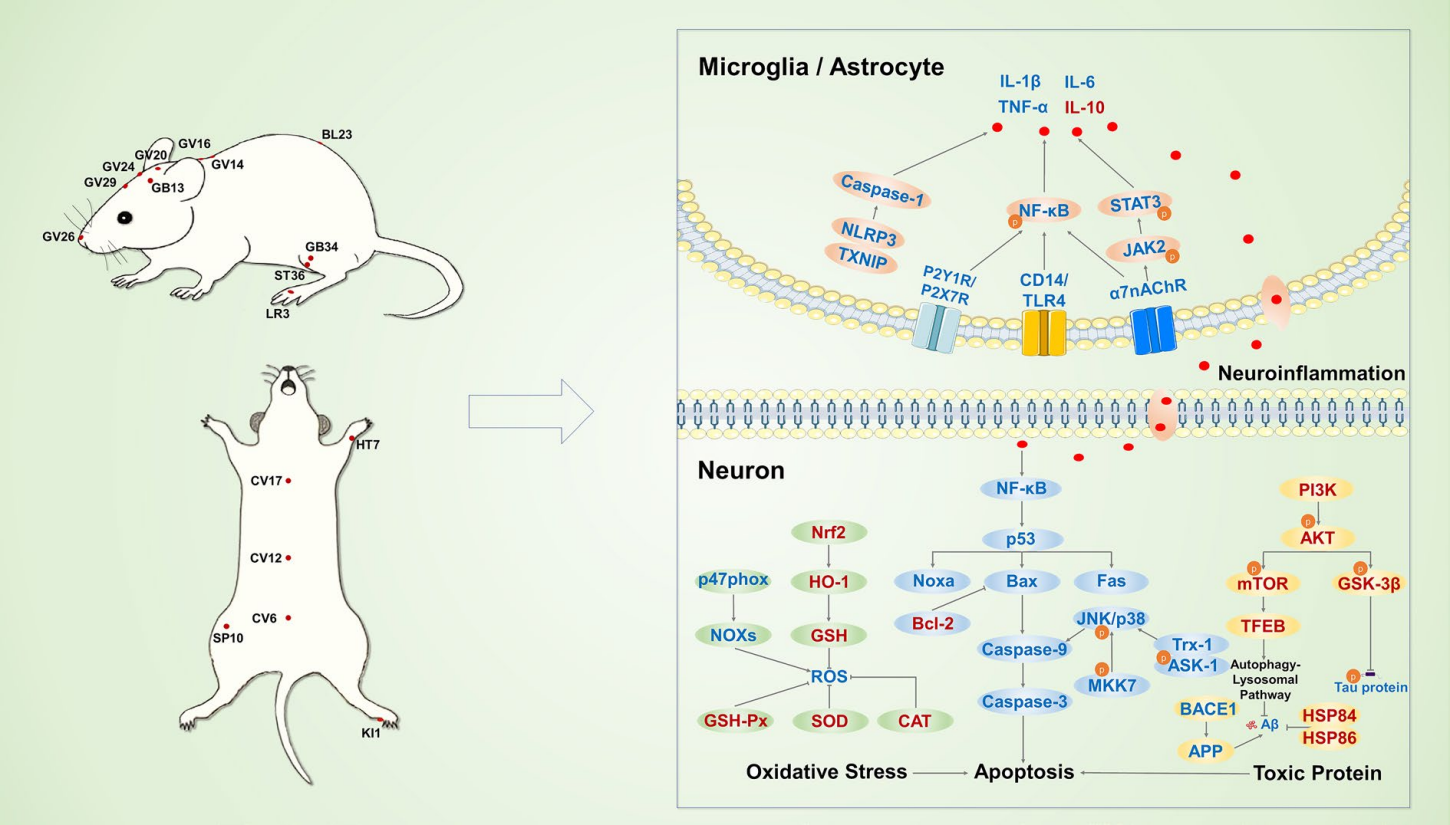
The neuroprotective mechanisms involved in acupoint stimulation for the treatment of cognitive impairment. The acupoints with neuroprotective effects for the treatment of cognitive impairment are marked in the image on the left. The neuroprotective mechanisms of acupoint stimulation are shown in the image on the right. Red characters, upregulated by acupoint stimulation; blue characters, downregulated by acupoint stimulation

**Fig. 3:**
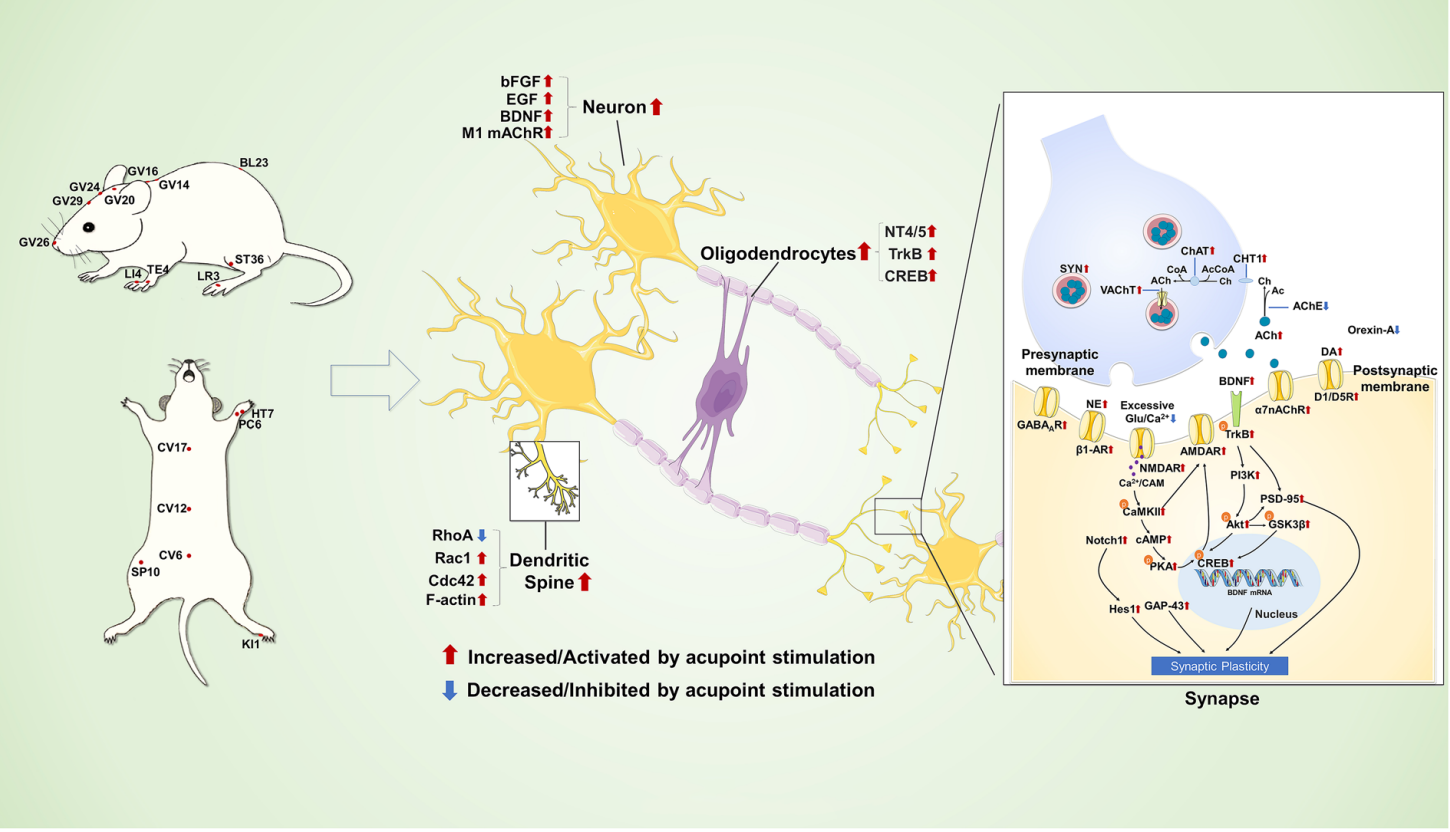
The neural regeneration and neuroplasticity mechanisms involved in acupoint stimulation for the treatment of cognitive impairment. The acupoints with neural regenerative and neuroplastic effects for the treatment of cognitive impairment are marked in the image on the left. Neural regeneration and neuroplasticity mechanisms of acupoint stimulation are shown in the image on the right

**Fig. 4:**
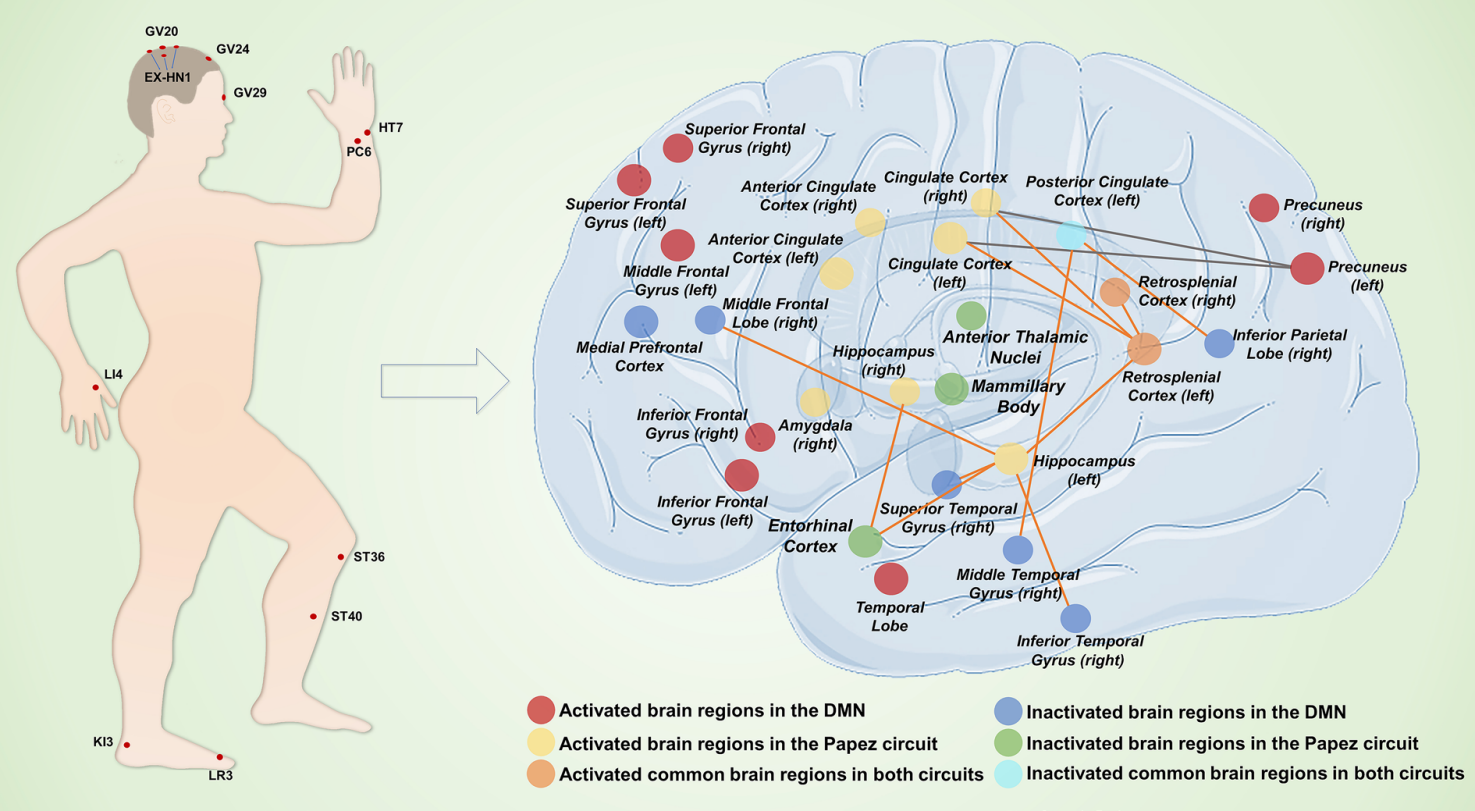
The neural circuit mechanisms involved in acupoint stimulation for the treatment of cognitive impairment. The acupoints used in neural circuit study for the treatment of cognitive impairment are marked in the image on the left. Neural circuit mechanisms of acupoint stimulation are shown in the image on the right

In conclusion, it has been demonstrated that acupoint stimulation can enhance spatial learning and memory function in cognitive impairment models. Specifically, MA and EA have been shown to directly inhibit cognition-related neuronal apoptotic pathways or to indirectly inhibit apoptosis by scavenging toxic proteins, battling oxidative stress, and reducing neuroinflammation, as shown in Fig. 2. In particular, acupoint stimulation can inhibit the production of Aβ and the phosphorylation of Tau protein and mitigate neuronal damage by promoting correct protein folding and regulating the autophagic degradation of toxic proteins. Acupoint stimulation prevents oxidative damage by regulating NOXs and Nrf2-related signaling pathways. Acupoint stimulation may also protect hippocampal neurons from neuroinflammatory damage by modulating the TLR4/MyD88/NF-κB, HMGB1/NF-κB, JAK2/STAT3, and P2 purinergic receptor signaling pathways. Additionally, acupoint stimulation can stimulate neural regeneration by increasing BDNF, bFGF, and EGF. It can also alter the synaptic structure and functional plasticity by controlling the BDNF/TrkB-PI3K/AKT/GSK-3β, cAMP/PKA/CREB signaling pathways, as well as glutamatergic, dopaminergic, noradrenergic, and cholinergic innervation (shown in Fig. 3). All of these effects of acupoint stimulation may contribute to the functional recovery of neurons in the hippocampal CA1, CA3, and DG regions, as well as the synaptic transmission in the PP-DG and Schaffer collaterals in the hippocampal trisynaptic circuit, to restore hippocampal activity, and FC with several brain regions in the Papez circuit and DMN. Moreover, acupoint stimulation altered functional activity and connectivity in the cingulate cortex, temporal lobe, and frontal lobe, among other locations (shown in Fig. 4). These pathways, as shown by molecular, cellular, and neuroimaging data, facilitate the amelioration of cognitive impairment by acupoint stimulation. This study illustrates the effectiveness of acupoint stimulation in treating cognitive impairment. However, additional high-quality research is necessary.

## Data Availability

Not applicable.

## Abbreviations

2VO: Bilateral common carotid artery occlusion
Ac: Acetic acid
AcCoA: Acetyl-coenzyme A
ACh: Acetylcholine
AChE: Acetylcholinesterase
AD: Alzheimer’s disease
ADL: Activity of daily living
AKT: Protein kinase B
ALFF: Amplitude of low-frequency fluctuations
AMPAR: α-Amino-3-hydroxy-5-methyl-4-isoazolpropionic acid receptors
APP: Amyloid precursor protein
ASK-1: Apoptosis signal-regulating kinase
Aβ: Amyloid β-peptide
Aβ1-42: Amyloid β-peptide 1–42
BACE1: Beta site amyloid precursor protein-cleaving enzyme 1
BDNF: Brain-derived neurotrophic factor
bFGF: Basic fibroblast growth factor
CaM: Calmodulin
CaMKII: Calcium/calmodulin-dependent protein kinase II
cAMP: 3′, 5′ Cyclic adenosine monophosphate
CAT: Catalase
CC: Corpus callosum
CCH: Chronic cerebral hypoperfusion
Cdc42: Cell division cycle 42
CG: Cingulate gyrus
Ch: Choline
ChAT: Choline acetyltransferase
CHT1: Choline transporter
CoA: Coenzyme A
CREB: CAMP response element-binding protein
CTSD: Cathepsin D
D1/D5R: Dopamine D1/D5 receptor
DA: Dopamine
DG: Dentate gyrus
DMN: Default mode network
DREADDs: Designer Receptors Exclusively Activated by Designer Drugs
EA: Electroacupuncture
EGF: Epidermal growth factor
FC: Functional connectivity
GABA_A_R: Type A gamma-aminobutyric acid chloride channel
GAP-43: Growth associated protein 43
Glu: Glutamate
GSH: Glutathione
GSH-Px: Glutathione peroxidase
GSK-3β: Glycogen synthase kinase-3beta
HMGB1: High mobility group box 1
HO-1: Heme oxygenase-1
HSP84: Heat shock protein 84
HSP86: Heat shock protein 86
I/R: Ischemia–reperfusion
IFG: Inferior frontal gyrus
IL-10: Interleukin-10
IL-1β: Interleukin-1beta
IL-6: Interleukin-6
iNOS: Inducible nitric oxide synthase
IPL: Inferior parietal lobe
JAK2: Janus kinase 2
JNK: C-Jun N-terminal kinase
LA: Laser acupuncture
LAMP1: Lysosome-associated membrane protein 1
LC3II: Microtubule-associated protein 1A/1B-light chain 3 II
LI/R: Limb ischemia–reperfusion
LTP: Long-term potentiation
M1 mAChR: M1-type muscarinic acetylcholine receptors
MA: Manual acupuncture
MCAO: Middle cerebral artery occlusion
MCI: Mild cognitive impairment
MDA: Malondialdehyde
MKK7: Mitogen-activated protein kinase kinase 7
MMSE: Mini-Mental State Examination
MTG: Middle temporal gyrus
MS: Medial septum
MTL: Medial temporal lobe
mTOR: Mammalian rapamycin target
MyD88: Myeloid differentiation primary response 88
NE: Norepinephrine
NF-κB: Nuclear factor-κB
NLRP3: NLR family pyrin domain-containing 3
NMDAR: N-methyl-d-aspartate receptor
NOXs: Nicotinamide adenine dinucleotide phosphate oxidases
Nrf2: Nuclear factor erythroid 2-related factor2
NSC: Neural stem cell
NT4/5: Neurotrophin 4/5
OLs: Oligodendrocytes
OPCs: Oligodendrocyte precursor cells
p: Phosphorylated
P2X7R: Purinergic P2X receptor 7
P2Y1R: Purinergic P2Y receptor 1
PCC: Posterior cingulate cortex
PCu: Precuneus
PD: Parkinson’s disease
PI3K: Phosphatidylinositol-3-kinase
PKA: Protein kinase A
PP-DG: Perforant pathway-dentate gyrus
PS1: Presenilin 1
PSD-95: Postsynaptic density protein 95
p-Tau: Phosphorylated Tau protein
Rac1: Small GTPase Ras-related C3 botulinum toxin substrate 1
RCTs: Randomized controlled trials
RhoA: Ras homolog gene family member A
ROS: Reactive oxygen species
RSC: Retrosplenial cortex
SAMP8: Senescence-accelerated mouse prone 8
SFG: Superior frontal gyrus
SOD: Superoxide dismutase
SQSTM1: Sequestosome 1
STAT3: Signal transducer and activator of transcription 3
SYN: Synaptophysin
TFEB: Transcription factor EB
TLR4: Toll-like receptor 4
TNF-α: Tumor necrosis factor alpha
TrkB: Tropomysin related kinase B
Trx-1: Thioredoxin-1
TrxR-1: Thioredoxin reductase-1
TXNIP: Thioredoxin interacting protein
VAChT: Vesicular acetylcholine transporter
VD: Vascular dementia
VDB: Vertical limb of the diagonal band of Broca
α7nAChR: α7-Nicotinic acetylcholine receptor
β1-AR: β1-Adrenergic receptors
